# Editorial: Integration of structural biology data in lead drug discovery and optimization

**DOI:** 10.3389/fmolb.2023.1145834

**Published:** 2023-02-01

**Authors:** Pietro Roversi, Gianluca Molla, Marco Nardini

**Affiliations:** ^1^ IBBA-CNR Unit of Milano, Institute of Agricultural Biology and Biotechnology, Milano, Italy; ^2^ Department of Molecular and Cell Biology, Leicester Institute of Structural and Chemical Biology, University of Leicester, Leicester, United Kingdom; ^3^ "The Protein Factory 2.0", Dipartimento di Biotecnologie e Scienze della Vita, Università degli Studi dell'Insubria, Varese, Italy; ^4^ Department of Biosciences, University of Milano, Milano, Italy

**Keywords:** lead drug discovery, fragment based drug discovery, integrative biology, virtual high-throughput screening, drug repositioning

Proteins, nucleic acids, and their complexes represent the vast majority of drug targets. The rational identification, design and optimization of a drug-lead compound targeting specific macromolecular sites is driven by the knowledge of the atomic interactions between the target itself and the small-molecule ligand under investigation. Of course, additional constraints besides the drug candidate’s molecular and chemical properties must be taken into consideration (*e.g.* bioavailability), but the knowledge of the molecular details of the lead-target interactions also plays a key role in the prediction and rationalisation of those properties. Thus, structural knowledge contributes both to the design and generation of initial lead compounds and to the medicinal chemistry process bridging from lead to final active pharmaceutical drug in the clinic.

Traditionally, macromolecular X-ray crystallography has been the experimental structural technique of choice for structure-based lead drug discovery (SBLDD), with Cryo-EM the latest addition to the structural biology toolbox (Cabral et al., 2022). Native Mass Spectrometry (nMS) now enters the same arena: the technique’s high sensitivity, simplicity, speed, wide dynamic range, low protein and ligand requirement, and the possibility of automation make nMS an integral component of the drug discovery pipeline, especially for primary screening (Gavriilidou et al.). When the dynamics of drug-target association matter, the great flexibility and adaptability of NMR provide for qualitative and quantitative insights at each point of the drug development process, aiding hit-identification and detection of weak binders, a paramount advantage in early stages of lead drug discovery (LDD) (Mureddu and Vuister).

Of course, advances in structural bioinformatics (*e.g.*, novel and/or improved data processing workflows or application of machine learning approaches) is just as important to drug discovery as the advances in the experimental techniques themselves. Progress on the experimental front will have to go hand in hand with changes in mindset both on the side of depositors and users of macromolecular models, as exemplified by developments in software and algorithms for X-ray Fragment Based Lead Discovery (FBLD) such as batch data processing methods, X-ray diffraction data analysis and presentation, modelling, refinement, deposition of structures (Pearce et al.) and automation of crystal polymorph assignment (Caputo et al.).

Determining the crystal structure of an enzyme-inhibitor complex may be considered the simplest example of integration of structural biology data in lead drug optimization, but the impact of such a simple experiment can often change the direction of the research trajectory. The study by Cianci et al. constitutes an example of an experimentally determined crystal structure correcting initial *in silico* predictions of a ligand-binding pose. Their crystal structure of human α-amino-β-carboxymuconate-ε-semialdehyde decarboxylase in complex with TES-1025 revealed unforeseen protein-ligand interactions, allowing the redefinition of the paradigmatic principles of the medicinal chemistry effort to improve potency and selectivity of the drug.

FBLD often affords the two-birds-with-one-scone simultaneous identification of novel lead scaffolds and previously unknown accessory allosteric drug binding sites on the target. The FBLD effort by Fiorillo et al., devoted to finding ligands of *T. brucei* trypanothione reductase, identified 12 new ligands, binding five different sites, two of which had not been exploited for inhibition previously. Crystal structures were also a key determinant in the success of the fragment screening campaigns carried out by Thomas et al. to inhibit *M. abscessus* and *M. tuberculosis* phosphopantetheine adenylyltransferase (PPAT). This study expands the chemical space of mycobacterial PPAT inhibition by discovering novel ligand-binding interactions in already known target sites, as well as previously undescribed ligand-induced cryptic sites.

When tackling the daunting challenge of discovering novel antibiotics, several parallel FBLD efforts targeting different previously neglected bacterial proteins hasten success. Arif et al. review computational approaches (such as structure modelling and assessment of the suitability of a protein as target for novel antibiotics) that informed the search of novel molecules binding previously untargeted pathogenic *Pseudomonas aeruginosa* proteins.

Structural information about conventional inhibitors can also serve as a starting point for more cutting-edge, unconventional molecules to be designed, as (Eli et al.) illustrate in their review on the identification of novel tubulin modulators and their delivery strategies. Novel classes of such molecules include PROTACs as well as light-sensitive compounds that can be activated with high spatial-temporal accuracy and therefore represent promising tools for precision-targeted chemotherapy.

Conversely, when a drug candidate affects the activity of its target in ways that cannot be detailed by experimental structural studies, integration of molecular dynamics (MD) and biochemical data can come to the rescue. The study on the effect of Fe^2+^ chelating compounds on the activity of human collagen lysyl hydroxylase by Scietti et al. reports on a fine balance between Fe^2+^-dependent enzymatic activity and Fe^2+^-induced self-inhibited enzyme conformations. The latter could not be captured by conventional structure-based approaches, but MD simulations were successful in rationalising the enzymatic activity data and boosting inhibitor development.

Last but not least, SBLDD can be speeded up by combining the power of *in silico* virtual high-throughput screening (VHTS) with the wealth of pharmacological information intrinsically carried by a drug repositioning approach. Antoniciello et al. successfully applied VHTS to a large number of known drugs already in clinic trials, checking their capability to inhibit the DNA-binding of the *Helicobacter pylori* HP1043 essential transcription factor. Three lead compounds that gave good activity *in vitro* turned out not to belong to the same chemical scaffold and to bind different protein sites, thus broadening the scope of the medicinal chemistry effort.

Overall, the contributions to this Research Topic highlight recent advances in structural biology, computational approaches, and high-throughput methods which have provided researchers with a huge amount of bewildering data and enabled novel approaches to data analysis and assessment. In this perspective, integrative structural biology converts structural information into chemical knowledge, feeding high quality experimental data into the drug development pipeline, thus supporting faster and more efficient delivery of drugs to the clinic.
